# Baseline CSF ferritin levels were associated with trajectories of depressive symptoms among older people without dementia

**DOI:** 10.3389/fnagi.2025.1516388

**Published:** 2025-10-22

**Authors:** Qing Wang, Xinwu Ye, Yongjian Lin

**Affiliations:** ^1^Department of Psychiatry, Wenzhou Seventh People’s Hospital, Wenzhou, Zhejiang, China; ^2^Department of Geriatric Psychiatry, Wenzhou Seventh People’s Hospital, Wenzhou, Zhejiang, China

**Keywords:** ferritin, iron, depressive symptoms, cluster analysis, longitudinal study

## Abstract

**Background:**

Previous studies have suggested a link between ferritin levels in the blood and depressive symptoms. However, no prior studies have investigated the association between cerebrospinal fluid (CSF) total ferritin, ferritin light chain, and ferritin heavy chain and longitudinal changes in depressive symptoms among older people without dementia.

**Methods:**

In this study, 543 older people without dementia were included, comprising 163 cognitively unimpaired (CU) participants and 380 participants with mild cognitive impairment (MCI). A non-parametric k-means longitudinal cluster analysis was performed to identify distinct trajectories of depressive symptoms, which were measured using the 15-item Geriatric Depression Scale (GDS-15) over a period of 5 years. Multinomial logistic regression models were used to examine the relationship between CSF total ferritin, ferritin light chain, and ferritin heavy chain levels and the trajectories of depressive symptoms, adjusting for potential covariates.

**Results:**

We identified three distinct trajectories of depressive symptoms: consistently low (trajectory 1, *n* = 364; mean age: 73 ± 7 years; percentage of females: 43%), moderately increasing (trajectory 2, *n* = 149; mean age: 72 ± 7 years; percentage of females: 43%), and rapidly increasing (trajectory 3, *n* = 30; mean age: 72 ± 8 years; percentage of females: 47%). Compared with trajectory 1, there was a significant relationship between membership in trajectory 3 and CSF total ferritin levels (OR = 0.04, 95% CI = 0.01 to 0.18, *p* < 0.001). Similarly, CSF ferritin light chain and heavy chain levels showed a similar pattern to that of CSF ferritin levels.

**Conclusion:**

Our study identified three distinct trajectories of depressive symptoms in older adults without dementia. We observed that lower CSF ferritin levels were associated with a higher likelihood of membership in the rapidly increasing symptom trajectory.

## Introduction

Depressive symptoms are common in older people and are often among the earliest behavioral changes observed in Alzheimer’s disease (AD; Blazer and Williams, 1980; Geda et al., 2008). Depressive symptoms may either increase an individual’s risk for developing AD or serve as a prodromal marker reflecting early pathological changes (Mejía et al., 2003; Panza et al., 2010; Li et al., 2011; Steenland et al., 2012; Dafsari and Jessen, 2020). Higher levels of depressive symptoms have been associated with key pathological hallmarks of AD, such as increased amyloid beta (Aβ; Babulal et al., 2016) and tau in the medial temporal lobe (Gatchel et al., 2017), even in cognitively normal older adults. Additionally, increases in depressive symptoms have been linked to deteriorating cognitive performance in older people with increased Aβ levels (Gatchel et al., 2019). Previous studies also highlight the important role of depressive symptomatology in financial capacity (Giannouli and Tsolaki, 2021a, 2021b; Giannouli et al., 2022). Therefore, it is crucial to identify potentially modifiable factors contributing to the development of depressive symptoms in older people without dementia.

Serum ferritin serves as a primary iron storage protein in the human body and is commonly used as a proxy to assess the body’s iron stores (Cook et al., 2003). Iron can initiate lipid peroxidation, which leads to altered membrane fluidity, inactivation of membrane-bound enzyme complexes, and ultimately results in edema, membrane disruption, and cell death (Lesnefsky, 1994). Several studies have explored the relationship between ferritin and depression, but the results are inconsistent. A previous study has reported that men with lower serum ferritin levels exhibit a higher prevalence of depressive symptoms among middle-aged adults (Yi et al., 2011). Conversely, other researchers have found a positive association between elevated serum ferritin levels and post-stroke depression (Zhu et al., 2016). No relationship between serum ferritin and depressive symptoms has also been reported previously (Su et al., 2016). One potential explanation for the inconsistencies is the use of ferritin levels from the blood rather than from the brain [i.e., cerebrospinal fluid (CSF)]. To the best of our knowledge, however, no prior studies have investigated the association of CSF total ferritin, ferritin light chain, and ferritin heavy chain with longitudinal changes in depressive symptoms among older people without dementia.

The aim of this study was to investigate the relationship between CSF total ferritin, ferritin light chain, and ferritin heavy chain levels and the trajectories of depressive symptoms over 5 years in a cohort of older individuals without dementia. This study aimed to address a gap in the current literature, as previous research has not fully explored the association between CSF ferritin and related biomarkers and the longitudinal changes in depressive symptoms. We hypothesized that individuals with lower baseline levels of CSF total ferritin, ferritin light chain, and ferritin heavy chain would be more likely to exhibit an increasing trajectory of depressive symptoms over time. Understanding this relationship could provide crucial insights into the biological mechanisms underlying depression and may inform the development of novel therapeutic interventions.

## Methods

### Alzheimer’s Disease Neuroimaging Initiative (ADNI)

Data used in the preparation of the current study were extracted from the ADNI database. The ADNI study was launched in 2003, with a primary goal of examining whether a variety of markers, such as serial MRI and positron emission tomography (PET) imaging markers, other fluid biological markers, and neuropsychological measures, can be integrated to track the cognitive changes of mild cognitive impairment (MCI) and mild AD dementia. Participants in the ADNI study are aged 55 to 90 years, fluent in either English or Spanish, and capable of participating in a longitudinal follow-up study. All participants should fall within the cognitive categories of normal cognition, MCI, and mild AD dementia. Recruitment procedures for the ADNI study have been described before (Aisen et al., 2024), and detailed inclusion criteria can be found online.^1^ Institutional review board approval was obtained at each participating ADNI site, and informed consent was obtained from each subject or authorized representative.

### Participants

Our study sample included subjects who met diagnostic criteria of normal cognition or MCI, had at least two assessments of depressive symptoms over a 5-year follow-up period, and had baseline CSF ferritin and related biomarkers available. There was a total of 543 participants, including 163 cognitively unimpaired (CU) participants and 380 participants with MCI. The criteria for CU included a Mini-Mental State Examination (MMSE; Folstein et al., 1975) score ranging from 24 to 30 and a Clinical Dementia Rating (CDR; Morris, 1993) score of 0. The criteria for MCI included an MMSE score ranging from 24 to 30, a CDR score of 0.5, a subjective memory complaint, objective memory impairment as assessed by the Wechsler Memory Scale Logical Memory II, and an essentially preserved ability to perform daily life activities (Aisen et al., 2024).

### Measurement of CSF ferritin and related biomarkers

CSF samples from the ADNI cohort were collected at baseline between 2005 and 2013 and subsequently stored at −80 °*C. Prior* to aliquoting, CSF samples were centrifuged at 1000 g for 10 s. Aliquot transfers were performed sequentially for each column (8 samples per column) using an 8-channel repeating pipette. The levels of total ferritin, ferritin light chain, and ferritin heavy chain in CSF were measured as part of proteomic assessments using SomaLogic’s SomaScan platform by the Neurogenomics and Informatics Center at Washington University. SomaLogic implemented initial standardization procedures to quantify these proteins (Timsina et al., 2022). Specifically, hybridization normalization was performed individually for each sample. Subsequently, aptamers were categorized into three distinct normalization groups—S1, S2, and S3—based on the signal-to-noise ratio observed in both technical replicates and samples. This classification was crucial to prevent the merging of aptamers with different protein signal intensities during further normalization steps. After this categorization, a median-based normalization approach was applied to address various assay-related inconsistencies, such as variations in protein concentration, pipetting, reagent concentration, and assay timing. Quality control included two criteria: (1) the maximum absolute difference between each aptamer’s scale factor and the plate median scale factor was < 0.5; (2) the median cross-plate coefficient of variation (CV) was ≤ 0.15. The CSF levels of total ferritin, ferritin light chain, and ferritin heavy chain are reported in relative fluorescence units (RFU). The values of these three markers were log-transformed prior to statistical analyses.

### Assessment of depressive symptoms

Depressive symptoms were measured using the 15-item Geriatric Depression Scale (GDS-15; Sheikh and Yesavage, 1986). GDS-15 scores range from 0 to 15, with higher scores representing more severe depressive symptoms. For this study, three subjects with baseline GDS-15 = 6 were excluded (all other subjects had GDS-15 scores ranging from 0 to 5, inclusive) since scoring ≥ 6 on the GDS-15 was categorized as manifesting symptoms of Major Depression (Marc et al., 2008; Mackin et al., 2012). This study used raw scores from the GDS-15 instead of z scores, as the former are more easily interpreted in clinical practice.

### Statistics

A non-parametric k-means longitudinal cluster analysis was performed to identify distinct trajectories of depressive symptoms over 5 years (Genolini et al., 2015). We conducted the longitudinal cluster analysis using the R package “kml” (Genolini et al., 2015). The repeatedly measured GDS-15 scores were modeled as our variable of interest. We built the clustering models for 1 to 8 trajectories and selected the 3-trajectory solution according to the Akaike Information Criterion (AIC), Bayesian Information Criterion (BIC), and the elbow method. In addition, we required that each trajectory must include more than 5% of the total sample. We conducted chi-square tests and analyses of variance to evaluate whether sample characteristics differed between the trajectories of depressive symptoms. More specifically, for the comparison of CSF total ferritin, ferritin light chain, and ferritin heavy chain levels, analyses of variance were used. If significant, follow-up multiple comparisons using pairwise t-tests were conducted. We performed multinomial logistic regression models to evaluate the relationship between CSF total ferritin, ferritin light chain, and ferritin heavy chain levels and the trajectories of depressive symptoms. The three trajectories were treated as the dependent variable, and CSF ferritin and related biomarkers were treated as independent variables. Covariates included age, gender, education, APOE4 status, MMSE score, history of hypertension, history of diabetes, and history of asthma. Three models were built separately for each CSF biomarker of interest (total ferritin, ferritin light chain, and ferritin heavy chain). All statistical analyses were performed using R software (www.r-project.org), and the significance level was set at *p* < 0.05.

## Results

### Trajectories of depressive symptoms

**Figure 1:**
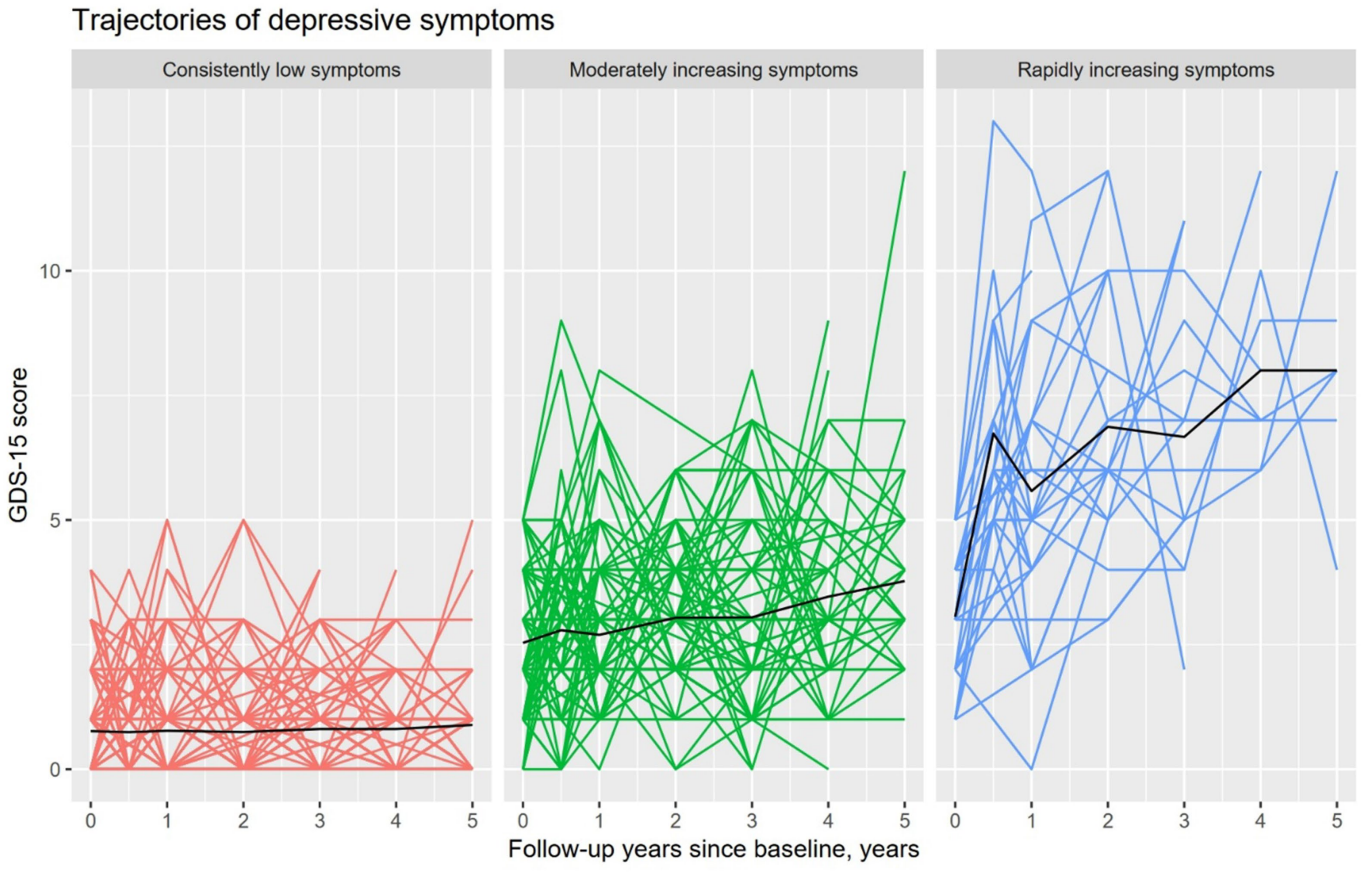
Trajectories of depressive symptoms based on GDS-15 scores among older people without dementia over a period of 5 years. Note: The colored lines represent individual depressive symptom trajectories over time. Specifically, red lines correspond to Trajectory 1 (“Consistently low symptoms”), green lines to Trajectory 2 (“Moderately increasing symptoms”), and blue lines to Trajectory 3 (“Rapidly increasing symptoms”). Due to several individuals following the same depressive trajectory, many lines overlap. The number of subjects in trajectories 1, 2, and 3 was 364, 149, and 30, respectively. GDS-15, 15-item Geriatric Depression Scale.

In order to identify distinct trajectories of depressive symptoms over 5 years, a non-parametric k-means longitudinal cluster analysis was conducted. The repeatedly measured GDS-15 scores were modeled as our variable of interest. We built the clustering models for 1 to 8 trajectories and selected the 3-trajectory solution according to the AIC, BIC, and the elbow method. In addition, we required that each trajectory must include more than 5% of the total sample. As demonstrated in Figure 1, our study sample was categorized into the following trajectories according to their trajectories of depressive symptoms: (1) trajectory 1 (*n* = 364, 67%), with consistently low levels of depressive symptoms over time; (2) trajectory 2 (*n* = 147, 27%), with moderately increasing levels of depressive symptoms; and (3) trajectory 3 (*n* = 30, 6%), with rapidly increasing levels of depressive symptoms. Therefore, we labeled the three trajectories as follows: consistently low, moderately increasing, and rapidly increasing symptoms.

### Comparison of baseline characteristics between depressive trajectories

Table 1 demonstrates the comparison of baseline characteristics between the three trajectories. There was a significant difference in baseline GDS-15 scores among the three trajectories, and all pairwise comparisons were significant. Regarding CSF total ferritin levels, trajectories 1 and 2 did not differ, while all other pairwise comparisons were significant. Specifically, trajectory 3 had lower levels of CSF total ferritin relative to trajectories 1 and 2. Similarly, trajectories 1 and 2 did not differ in CSF ferritin light chain or heavy chain levels, while all other pairwise comparisons were significant. There were no significant differences in other variables among the trajectories (all *p* > 0.05; Table 1).

**Table 1:** Summary of baseline characteristics.

Characteristic	Trajectory 1 *N* = 364	Trajectory 2 *N* = 149	Trajectory 3 *N* = 30	Effect size	*p*-value
Age, years	73 (7)	72 (7)	72 (8)	η^2^ = 0.004	0.4
Education, years	16 (3)	16 (3)	16 (4)	η^2^ = 0.004	0.4
Gender				Cramér’s V = 0.02	>0.9
Male	206 (57%)	85 (57%)	16 (53%)		
Female	158 (43%)	64 (43%)	14 (47%)		
APOE4 status				Cramér’s V = 0.09	0.10
APOE4-	215 (59%)	73 (49%)	18 (60%)		
APOE4+	149 (41%)	76 (51%)	12 (40%)		
MMSE	28 (2)	28 (2)	28 (2)	η^2^ = 0.008	0.12
Hypertension	151 (41%)	77 (52%)	15 (50%)	Cramér’s V = 0.09	0.091
Diabetes	27 (7.4%)	16 (11%)	5 (17%)	Cramér’s V = 0.08	0.12
Asthma	23 (6.3%)	13 (8.7%)	0 (0%)	Cramér’s V = 0.09	0.2
GDS-15 score	1 (1)	3 (1)^a^	3 (1)^a^^,^^b^	η^2^ = 0.44	<0.001
Number of assessments of GDS-15	4.9 (1.3)	4.89 (1.48)	4.33 (1.37)	η^2^ = 0.009	0.1
CSF biomarkers, log RFU					
Total ferritin	9.27 (0.26)	9.24 (0.24)	9.07 (0.23)^a^^,^^b^	η^2^ = 0.03	<0.001
Ferritin light chain	9.15 (0.27)	9.11 (0.25)	8.94 (0.25)^a^^,^^b^	η^2^ = 0.03	<0.001
Missing, n	0	1	0		
Ferritin heavy chain	6.88 (0.15)	6.89 (0.16)	6.79 (0.15)^a^^,^^b^	η^2^ = 0.02	0.012
Missing, n	8	2	0		

Continuous variables are summarized as Mean (SD), and categorical variables are summarized as n (%). Continuous variables were compared using ANOVA tests, categorical variables were analyzed with Chi-squared tests, and the history of diabetes and asthma was compared using Fisher’s exact tests. APOE4, Apolipoprotein E ε4; MMSE, Mini-Mental State Examination; GDS-15, 15-item Geriatric Depression Scale; RFU, Relative Fluorescence Units.

a *p* < 0.05 compared with trajectory 1.

b *p* < 0.05 compared with trajectory 2.

### CSF ferritin and related biomarkers in the three depressive trajectories

**Figure 2:**
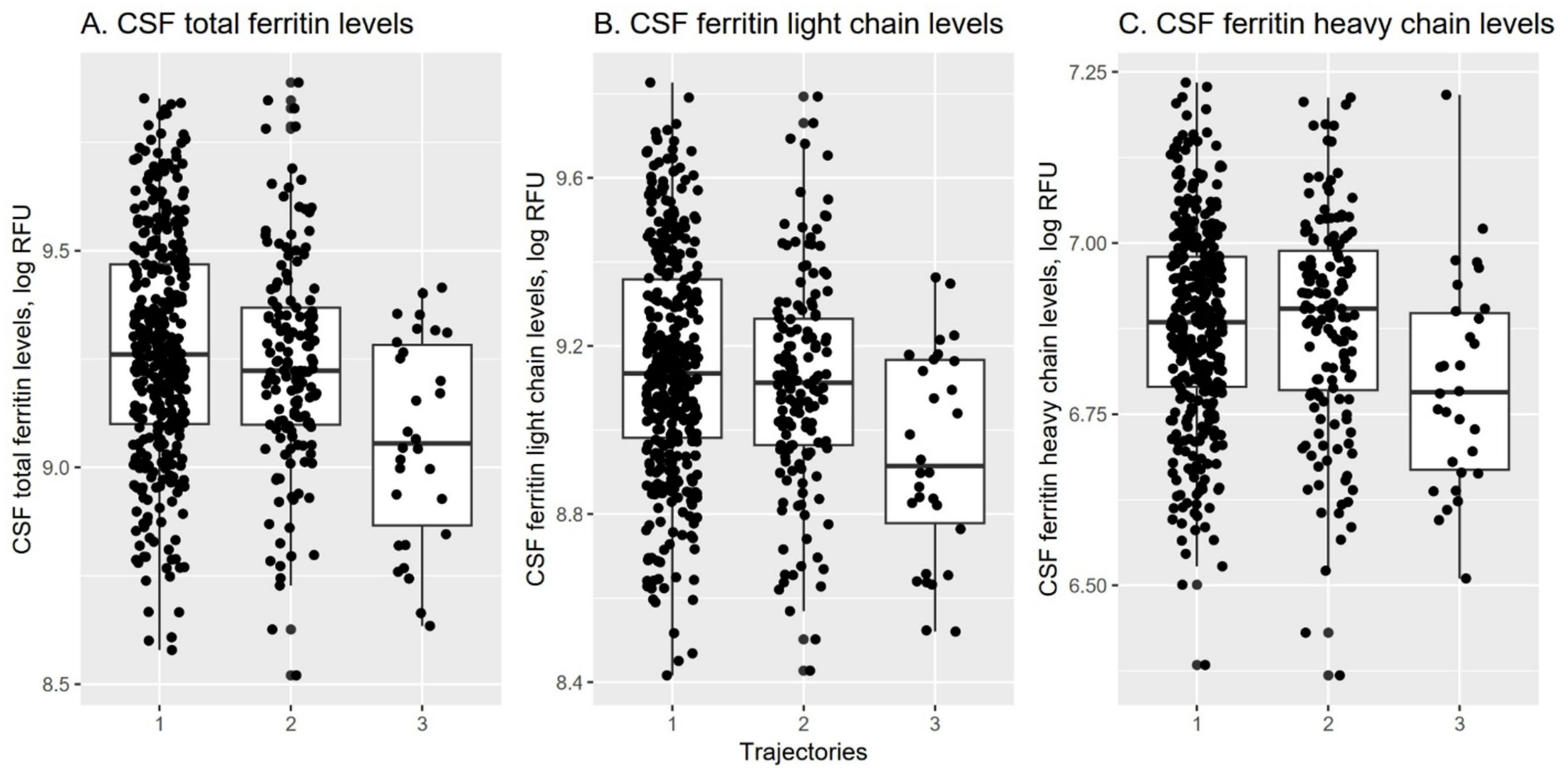
CSF ferritin and related biomarkers in the three trajectories. **(A)** Shows the levels of CSF total ferritin across the three trajectories, **(B)** shows the levels of CSF ferritin light chain, and **(C)** shows the levels of CSF ferritin heavy chain. For all CSF ferritin biomarkers, the trajectories 1 and 2 did not differ, while all other pairwise comparisons were significant.

To compare levels of CSF ferritin and related biomarkers among the three trajectories, ANOVAs and *post hoc* pairwise t-tests were conducted. As shown in Figure 2, there were significant differences in CSF ferritin and related variables among the three trajectories. Specifically, Figure 2A demonstrates the levels of CSF total ferritin among the three trajectories, Figure 2B shows the levels of CSF ferritin light chain, and Figure 2C illustrates the levels of CSF ferritin heavy chain. For all CSF ferritin biomarkers, the trajectories 1 and 2 did not differ, while all other pairwise comparisons were significant. Specifically, trajectory 3 had lower levels of CSF total ferritin, ferritin light chain, and ferritin heavy chain compared to trajectories 1 and 2 (Table 1; Figure 2).

### Summaries of multinomial logistic regression models

To evaluate the relationships between trajectory membership and CSF ferritin and related biomarkers, while considering potential covariates, multinomial logistic regression was employed. As shown in Table 2, the multinomial logistic regression model with CSF total ferritin levels as the predictor of interest suggested that, compared with trajectory 1, there was a significant relationship between membership in trajectory 3 and CSF total ferritin levels (OR = 0.04, 95% CI = 0.01 to 0.18, *p* < 0.001). In contrast, no significant association was observed between membership in trajectory 2 and CSF total ferritin levels (OR = 0.58, 95% CI = 0.26 to 1.28, *p* = 0.2). Likewise, as shown in Table 3, the model suggested that, compared with trajectory 1, there was a significant relationship between membership in trajectory 3 and CSF ferritin light chain levels (OR = 0.04, 95% CI = 0.01 to 0.21, *p* < 0.001). In contrast, no significant association was observed between membership in trajectory 2 and CSF ferritin light chain levels (OR = 0.54, 95% CI = 0.25 to 1.15, *p* = 0.11). As shown in Table 4, the model suggested that, compared with trajectory 1, there was a significant relationship between membership in trajectory 3 and CSF ferritin heavy chain levels (OR = 0.02, 95% CI = 0.00 to 0.27, *p* = 0.003). In contrast, no significant association was observed between membership in trajectory 2 and CSF ferritin heavy chain levels (OR = 1.17, 95% CI = 0.31 to 4.37, *p* = 0.8).

**Table 2:** Multinomial logistic regression model with CSF total ferritin levels as the predictor of interest.

Characteristic	Trajectories 2 vs 1			Trajectories 3 vs 1		
	OR	95% CI	*p*-value	OR	95% CI	*p*-value
Age	0.98	0.96, 1.01	0.3	0.99	0.93, 1.04	0.6
Education	0.97	0.90, 1.04	0.4	1.03	0.89, 1.19	0.7
Gender						
Male	—	—		—	—	
Female	0.92	0.61, 1.39	0.7	1.04	0.46, 2.37	>0.9
APOE4 status						
APOE4-	—	—		—	—	
APOE4+	1.42	0.95, 2.13	0.087	1.03	0.46, 2.33	>0.9
MMSE	0.92	0.82, 1.03	0.2	0.82	0.65, 1.03	0.082
Hypertension	1.44	0.96, 2.14	0.075	1.23	0.56, 2.72	0.6
Diabetes	1.31	0.67, 2.57	0.4	2.49	0.81, 7.67	0.11
Asthma	1.35	0.65, 2.80	0.4	0.00	0.00, 0.00	<0.001
CSF ferritin levels	0.58	0.26, 1.28	0.2	0.04	0.01, 0.18	<0.001

OR, Odds Ratio; CI, Confidence interval; APOE4, Apolipoprotein E ε4; MMSE, Mini-Mental State Examination.

**Table 3:** Multinomial logistic regression model with CSF ferritin light chain levels as the predictor of interest.

Characteristic	Trajectories 2 vs 1			Trajectories 3 vs 1		
	OR	95% CI	*p*-value	OR	95% CI	*p*-value
Age	0.98	0.95, 1.01	0.3	0.99	0.93, 1.04	0.6
Education	0.96	0.90, 1.04	0.3	1.03	0.89, 1.19	0.7
Gender						
Male	—	—		—	—	
Female	0.93	0.61, 1.40	0.7	1.06	0.47, 2.39	0.9
APOE4 status						
APOE4-	—	—		—	—	
APOE4+	1.40	0.94, 2.10	0.10	1.01	0.45, 2.28	>0.9
MMSE	0.92	0.82, 1.03	0.15	0.82	0.65, 1.02	0.078
Hypertension	1.45	0.97, 2.17	0.067	1.25	0.57, 2.76	0.6
Diabetes	1.31	0.67, 2.58	0.4	2.40	0.78, 7.39	0.13
Asthma	1.35	0.65, 2.80	0.4	0.00	0.00, 0.00	<0.001
CSF ferritin light chain levels	0.54	0.25, 1.15	0.11	0.04	0.01, 0.21	<0.001

OR, Odds Ratio; CI, Confidence interval; APOE4, Apolipoprotein E ε4; MMSE, Mini-Mental State Examination.

**Table 4:** Multinomial logistic regression model with CSF ferritin heavy chain levels as the predictor of interest.

Characteristic	Trajectories 2 vs 1			Trajectories 3 vs 1		
	OR	95% CI	*p*-value	OR	95% CI	*p*-value
Age	0.98	0.95, 1.01	0.2	0.98	0.92, 1.03	0.4
Education	0.96	0.89, 1.03	0.3	1.02	0.89, 1.19	0.7
Gender						
Male	—	—		—	—	
Female	0.90	0.59, 1.35	0.6	1.14	0.51, 2.54	0.8
APOE4 status						
APOE4-	—	—		—	—	
APOE4+	1.30	0.87, 1.95	0.2	0.79	0.35, 1.77	0.6
MMSE	0.92	0.82, 1.04	0.2	0.81	0.65, 1.02	0.073
Hypertension	1.42	0.95, 2.13	0.083	1.34	0.61, 2.93	0.5
Diabetes	1.27	0.65, 2.49	0.5	2.19	0.73, 6.56	0.2
Asthma	1.34	0.64, 2.77	0.4	0.00	0.00, 0.00	<0.001
CSF ferritin heavy chain levels	1.17	0.31, 4.37	0.8	0.02	0.00, 0.27	0.003

OR, Odds Ratio; CI, Confidence interval; APOE4, Apolipoprotein E ε4; MMSE, Mini-Mental State Examination.

### Supplementary analyses

First, we compared CSF Aβ42 and p-tau181 levels across the three depressive trajectories using ANOVA, and found that there were significant differences (Supplementary Table 1). *Post-hoc* pairwise *t*-tests with FDR correction showed that trajectory 1 had higher levels of CSF Aβ42 than trajectory 2, with no other significant differences. For CSF p-tau181, trajectory 2 had significantly higher levels than trajectories 1 and 3, while no other pairwise comparisons were significant.

Second, we compared the percentage of MCI subjects across the three depressive trajectories using Pearson’s Chi-squared test, and found there were significant differences (Supplementary Table 2). *Post-hoc* pairwise Pearson’s Chi-squared tests with FDR correction suggested that trajectories 2 and 3 had significantly higher percentage of MCI cases than trajectory 1, while no significant differences was found between trajectory 2 and 3.

Third, we calculated the ratios of ferritin light chain to total ferritin (light/total), ferritin heavy chain to total ferritin (heavy/total), and light chain to heavy chain (light/total), and compared these across the three depressive trajectories using ANOVA. Significant differences were observed for the heavy/total and light/heavy ratios, but not for the light/total ratio (Supplementary Table 3). However, *post-hoc* pairwise t-tests with FDR correction showed no significant pairwise comparisons for any ratio.

Fourth, we compared two markers of oxidative stress, including superoxide dismutase 1 (SOD1) and glutathione peroxidase 1 (GPx1), across the three depressive trajectories using ANOVA. Significant differences were found for SOD1, but not for GPx1 (Supplementary Table 4). Post hoc pairwise t-tests with FDR correction showed that trajectory 3 had significantly lower levels of SOD1 than trajectory 1, while no other pairwise comparisons were significant (i.e., trajectory 1 vs. 2 or trajectory 2 vs. 3).

## Discussion

To the best of our knowledge, this is the first study to examine the association of CSF total ferritin, ferritin light chain, and ferritin heavy chain with longitudinal changes in depressive symptoms among older people without dementia. In this study, we identified three distinct trajectories of depressive symptoms among older people without dementia: consistently low (trajectory 1), moderately increasing (trajectory 2), and rapidly increasing symptoms (trajectory 3). Additionally, we observed that lower levels of CSF total ferritin were associated with a higher likelihood of developing rapidly increasing symptoms compared to consistently low symptoms. Similarly, CSF ferritin light chain and heavy chain levels showed a similar pattern to that of CSF total ferritin levels.

The identification of three distinct trajectories of depressive symptoms—consistently low, moderately increasing, and rapidly increasing—is in line with previous findings that suggest the heterogeneity of depressive symptoms among older people (Kaup et al., 2016; Mirza et al., 2016; Barca et al., 2017; Petkus et al., 2024). This heterogeneity underscores the need for individualized approaches to the management and prevention of depression in older adults. Individuals in trajectory 1 exhibit stable and low levels of depressive symptoms over time. This group may represent individuals who have protective factors or effective coping mechanisms that help maintain their mental health. The second trajectory indicates a gradual increase in depressive symptoms, while the third trajectory shows a rapid increase in symptoms. Trajectory 3 is at the highest risk of developing clinically significant depression and may require more intensive monitoring and intervention. Future studies are warranted to replicate these findings in larger and more diverse populations to validate the trajectories.

Our study found that lower levels of CSF ferritin, including ferritin light chain and heavy chain levels, were associated with a higher likelihood of membership in the rapidly increasing symptom trajectory (trajectory 3) compared to the consistently low symptom trajectory (trajectory 1). This finding aligns with previous studies suggesting a link between iron metabolism and depression (Vahdat Shariatpanaahi et al., 2007; Yi et al., 2011; Wang et al., 2021; Ciulei et al., 2023; Leung and Kyung, 2024). Several potential mechanisms may explain this relationship. First, iron is essential for the synthesis and metabolism of neurotransmitters such as serotonin, norepinephrine, and dopamine (Parks and Wharton, 1989). Lower CSF ferritin levels, which indicate reduced iron stores, may impair these processes, leading to neurotransmitter imbalances and contributing to the development of depressive symptoms. Second, iron metabolism affects the regulation of oxidative stress and inflammation (Cornelissen et al., 2019). Lower levels of ferritin may result in increased oxidative stress and neuroinflammation (Cornelissen et al., 2019; Jiang et al., 2025), both of which have been implicated in the pathogenesis of depression (Maes et al., 2025; Teixeira et al., 2025). The observed association between lower CSF ferritin levels and rapidly increasing depressive symptoms may be mediated by these pathways. Third, iron is also necessary for proper brain function, including neuroplasticity and synaptogenesis (Muñoz and Humeres, 2012b). Reduced iron availability, as indicated by lower ferritin levels, may negatively affect these processes, potentially contributing to the development of depressive symptoms (Leung and Kyung, 2024; Muñoz and Humeres, 2012a). Our findings are consistent with some (Yi et al., 2011), but not all (Su et al., 2016; Zhu et al., 2016), previous studies that examined the association between blood ferritin and depressive symptoms. For example, consistent with our findings, a previous study found that men with lower serum ferritin levels exhibit a higher prevalence of depressive symptoms among middle-aged adults (Yi et al., 2011). However, Zhu and colleagues suggested a positive relationship between elevated serum ferritin levels and post-stroke depression (Zhu et al., 2016). This inconsistency may be due to differences in study samples, for example, between ischemic stroke patients and older people without dementia. Additionally, Su and colleagues did not find a relationship between serum ferritin and depressive symptoms in a cross-sectional study (Su et al., 2016). This may suggest that the relationship between serum ferritin and depressive symptoms is not readily captured by a cross-sectional study design. One other potential explanation for the inconsistencies is the use of ferritin levels from the blood rather than from the brain (i.e., CSF). These inconsistencies may be attributed to several other factors, such as participant age, cognitive status, depression assessment tools, frequency of symptom assessment, and statistical methods. Further studies are warranted to examine the association between ferritin and depressive symptoms.

This study has several limitations. First, the observational nature of the data limits our ability to establish causality, and experimental studies are needed to confirm the temporal relationship between CSF ferritin levels and depressive symptom trajectories. Second, the generalizability of our findings may be constrained by the specific characteristics of the study population. Further research in more diverse populations is needed to validate these results. Third, this study should be considered exploratory in nature. Future studies with a larger sample size, especially in Trajectory 3, are necessary to validate the results. Fourth, because the ADNI focuses on cognitive decline in individuals with MCI and mild AD dementia, it does not include standardized diagnostic criteria for clinical depression. Therefore, in this study, we evaluated depressive symptomatology using the GDS-15 rather than conducting a formal assessment of clinical depression. Fifth, the current study lacks longitudinal measurements of CSF total ferritin, ferritin light chain, and ferritin heavy chain. Future studies should include such measurements to better understand their trajectories over time. Sixth, it would be interesting to examine the associations between plasma levels of total ferritin, ferritin light chain, and ferritin heavy chain and changes in depressive symptoms over time. Further studies are needed to address these questions.

In conclusion, our study identified three distinct trajectories of depressive symptoms in older adults without dementia and found that lower CSF ferritin levels were associated with a higher likelihood of rapidly increasing depressive symptoms. These findings underscore the importance of considering iron metabolism in the pathophysiology of depression and highlight the need for personalized approaches to the management and prevention of depression in older adults. Further research is needed to fully understand the mechanisms underlying this association and to develop effective therapeutic strategies.

## Data Availability

Publicly available datasets were used in the current study. This data can be found at: the Alzheimer’s Disease Neuroimaging Initiative (ADNI) database (adni.loni.usc.edu).

## Footnotes

^1^ https://adni.loni.usc.edu/wp-content/themes/freshnews-dev-v2/documents/clinical/ADNI-1_Protocol.pdf

## Alzheimer’s Disease Neuroimaging Initiative (ADNI)

Data used in the preparation of this article were obtained from the Alzheimer’s Disease Neuroimaging Initiative (ADNI) database (adni.loni.usc.edu). As such, the investigators within the ADNI contributed to the design and implementation of ADNI and/or provided data but did not participate in the analysis or writing of this report. A complete listing of ADNI investigators can be found at: http://adni.loni.usc.edu/wp-content/uploads/how_to_apply/ADNI_Acknowledgement_List.pdf.
