# Supplementary material for: Baseline CSF ferritin levels were associated with trajectories of depressive symptoms among older people without dementia
Source: Front Aging Neurosci. 2025 Oct 22;17:1516388. doi: 10.3389/fnagi.2025.1516388 (PMC12587325; doi:10.3389/fnagi.2025.1516388)
Supplement: Supplementary file 1 [file Table_1.DOCX]

**Supplementary Table 1. Comparisons of CSF AD biomarkers across the three trajectories**

| **Characteristic** | **Trajectory 1**  N = 364 | **Trajectory 2**  N = 149 | **Trajectory 3**  N = 30 | **p-value** |
| --- | --- | --- | --- | --- |
| CSF Aβ42, pg/ml | 1,073 (447) | 958 (439) ^a^ | 1,035 (433) | 0.035 |
| CSF ptau181, pg/ml | 26 (14) | 29 (15) ^a^ | 22 (10) ^b^ | 0.009 |
| Notes: | | | | |

^a^ p<0.05 compared with trajectory 1

^b^ p<0.05 compared with trajectory 2

**Supplementary Table 2. Comparisons of percentage of MCI cases across the three trajectories**

| **Characteristic** | **Trajectory 1**  N = 364 | **Trajectory 2**  N = 149 | **Trajectory 3**  N = 30 | **p-value** |
| --- | --- | --- | --- | --- |
| Cognitive status |  |  |  | <0.001 |
| CU | 144 (40%) | 16 (11%) | 3 (10%) |  |
| MCI | 220 (60%) | 133 (89%) ^a^ | 27 (90%) ^a^ |  |

Notes:

^a^ p<0.05 compared with trajectory 1

^b^ p<0.05 compared with trajectory 2

**Supplementary Table 3. Comparisons of three ratios across the three trajectories**

| **Characteristic** | **Trajectory 1**  N = 364 | **Trajectory 2**  N = 149 | **Trajectory 3**  N = 30 | **p-value** |
| --- | --- | --- | --- | --- |
| Light/total | 0.987 (0.005) | 0.987 (0.005) | 0.987 (0.004) | 0.5 |
| Missing, n | 0 | 1 | 0 |  |
| Heavy/total | 0.743 (0.017) | 0.746 (0.017) | 0.750 (0.016) | 0.039 |
| Missing, n | 8 | 2 | 0 |  |
| Light/heavy | 1.33 (0.03) | 1.32 (0.03) | 1.32 (0.03) | 0.027 |
| Missing, n | 8 | 3 | 0 |  |

Notes: No significant pairwise comparisons were found across the three trajectories with FDR correction.

**Supplementary Table 4. Comparisons of two markers of oxidative stress across the three trajectories**

| **Characteristic** | **Trajectory 1**  N = 364 | **Trajectory 2**  N = 149 | **Trajectory 3**  N = 30 | **p-value** |
| --- | --- | --- | --- | --- |
| SOD1 | 344 (28) | 342 (26) | 331 (27) ^a^ | 0.042 |
| Missing, n | 14 | 9 | 1 |  |
| GPx1 | 124 (12) | 125 (13) | 121 (13) | 0.4 |
| Missing, n | 37 | 12 | 5 |  |

Note: ^a^ p<0.05 compared with trajectory 1
